# Employment, Studies and Feelings: Two to Nine Years After a Personalized Program of Cognitive Remediation in Psychiatric Patients

**DOI:** 10.3389/fpsyt.2020.00609

**Published:** 2020-07-03

**Authors:** Isabelle Amado, Mona Moualla, Julia Jouve, Lindsay Brénugat-Herné, David Attali, Dominique Willard, Bérangère Rigaut, Brigitte Malangin, Laurence Kern, Clementine Meyniel, Raphaël Gaillard, Marion Plaze, Florence Perquier, Morvan Yannick

**Affiliations:** ^1^ Groupe Hospitalier Universitaire Paris Psychiatrie et Neurosciences, Sainte Anne Hospital, Paris, France; ^2^ Psychiatry and Neuroscience Center, National Resource Center for Cognitive Remediation and Psychosocial Rehabilitation (C3RP), Paris, France; ^3^ Inserm U 1266, Paris, France; ^4^ Cundill Center for Child and Youth Depression, Université de Paris, Paris, France; ^5^ Laboratoire, CeSRM, UFR STAPS, Université Paris Nanterre, Nanterre, France; ^6^ Laboratoire CLIPSYD, EA4430, Université Paris Nanterre, Nanterre, France; ^7^ Center for Addiction and Mental Health, Toronto, ON, Canada

**Keywords:** cognitive remediation, long term outcome, employment, rehabilitation

## Abstract

Employment rate in psychiatry is around 10 to 30%. Cognitive remediation (CR) associated with psychosocial rehabilitation shows good functional outcomes, with a high level of satisfaction in participants provided by tailored CR. However, few studies looked at the long-term outcome in participants who experienced such a program. This retrospective survey examines the outcome of persons having psychiatric diseases 2 to 9 years after being treated with a personalized CR program. The survey included 12 domains with questions relevant to work, studies, before CR (T1) and at the moment of the survey (T2), questions about housing, relatedness, familiar relationships and daily activities at T2. Finally, a narrative interview was included to express feelings of the participants about CR. Sixty-six participants completed the survey, and were treated with neurocognitive or social cognition programs. Their diagnosis was: schizophrenia (80.3%), neurodevelopment disorder (autism as well as genetic or metabolic disease with psychiatric expression) (15.2%) and bipolar disorder (4.5%). The comparison between T1 and T2 showed significant difference for job employment (P < 0.001), even for competitive jobs (p < 0.007), for performing studies (p = 0.033), for practicing a physical activity (0.033) or reading (0.002). Outcome was also examined in reference to the delay from CR to highlight changes in patient characteristics and service delivery over the years. Hence, the total sample was split in two subgroups: CR delivered in 2009–2013 (n = 37); CR delivered in 2014–2016 (n = 29). While in the former group more participants were working (p = 0.037), in the latter group, which was younger (p = 0.04), more participants were studying (p = 0.02). At T2, a majority of persons experienced no relapse, three years (79.1%) to 8 years (56.8%) after CR, when referring to the anamnesis. Concerning subjective perception of CR, participants expressed feelings concerning positive impact on clarity of thought, on cognitive functions, self-confidence, perceiving CR as an efficient help for work and studies. To conclude, even long years after a personalized CR program, good benefits in terms of employment or studies emerge when compared to the status before CR, with good determinants for recovery in terms of leisure or physical activity practice.

## Introduction

Employment rate for people with severe mental illness is only around 10 to 30% (1–3). The literature mentions that poor cognitive functioning affects vocational outcomes in patients with severe mental illness, and even for those receiving vocational rehabilitation services (4–9). The programs for cognitive remediation (CR) are generally categorized as manual-task training or computer-assisted training, and concern neurocognition as well as social cognition. CR improves cognitive functioning in schizophrenia (10, 11), in autistic spectrum disorders (12), bipolar disorders (13) or in complex neurodevelopmental disorders (14). This psychosocial therapy provides benefits on symptoms and improves self-esteem (4, 15, 16) as well as self-efficiency to achieve personal goals (17), with maintained long term benefits (12, 18). Meta-analyses demonstrated that CR associated with adjunctive psychosocial rehabilitation shows stronger effects on functional outcomes compared to programs not associated to rehabilitation (8, 10, 12). Psychosocial rehabilitation includes psychosocial therapies such as psychoeducation, for users and care-givers, cognitive behavior therapy or psychosocial skills intervention. These therapies can facilitate the transfer of benefits acquired during CR programs to everyday life (19). However, the vast majority of Research done around CR programs focused on internal validity rather than trying to extent findings on real world context (20). Recently, a CR program, called “Cognitive Remediation to Promote Recovery” (CR2PR), has been developed in 16 clinics in New York for patients with serious mental illness (21). The principle of CR2PR program insisted on the point that “cognitive remediation programs had to be delivered tied to overall recovery goals” to increase the impact on functional outcome (11, 22). The results in this study after the participation of the users averaged 90.5%. Also, with an average number of 138 patients across the clinical sites, 40% of the users self-reported a high level of satisfaction with the service, and 96.9% qualified it as an excellent or good experience. Most patients found that CR improved cognition, and for 90% CR helped them to deal more effectively with situations at home, school, work, or with friends (21). Furthermore, Medalia et al. (19), as well as Seccomandi et al. (23) suggested to provide a “personalized medicine’ with tailored medical intervention for CR, bringing an answer to the fact that around 25% of the patients do not improve after CR.

**Figure 1:**
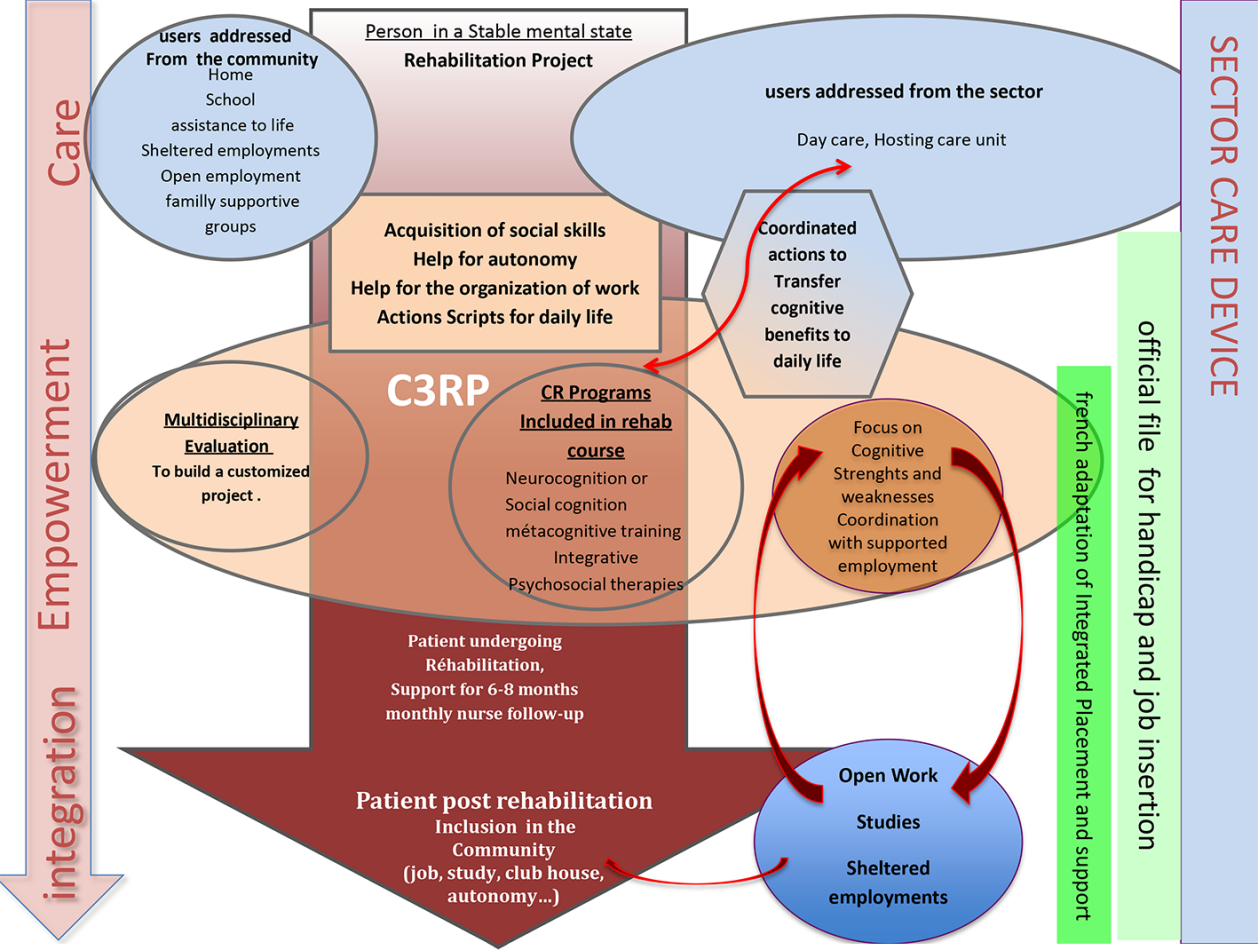
The stepping stone model for empowerment and recovery.

The French Center for Cognitive Remediation and Psychosocial Rehabilitation (C3RP) was created in 2009, to deliver personalized CR programs as well as psychosocial rehabilitation course for persons with schizophrenia, autism or complex neurodevelopmental disorders (psychiatric troubles with genetic or metabolic diseases). These programs are delivered in a patient-centered approach to provide services responsive to patients’ preferences and wishes, focusing on the cognitive profile of each patient, rather than on his diagnosis (22). Moreover, CR programs are fully personalized and delivered in coordination with the French care-units attached to the “sectors teams” associated to the patient residential home, or attached to the private medical practitioner in charge of the clinical follow-up. This coordination is efficient throughout the program. Also, the C3RP must organize an efficient relay with the unit that will accompany the user throughout his professional insertion or help to concretize his rehabilitation project. To see if a user is eligible to a CR program the practitioner must determine a core set of four characteristics: 1) if the user is clinically stable, 2) if the treatment is stable, with no sedative compounds (such as anxiolytics) delivered during the day, and well adjusted (for at least 1 month) 3) if the user is fully engaged to participate to CR programs and 4) if there is a concrete idea of a rehabilitation project. The CR schedule must be timely coordinated to the rehabilitation project, in order to act as a “stepping stone” and to increase the chance for the project to be successful (see **Figure 1**). The concrete phase of the project must begin 6 to 8 months after the end of the CR (mean duration for the maintain of cognitive benefits) (24). Whatever the type of CR program, neurocognitive or social cognition, the nodal point is the link between CR and transfer to daily life (19). For neurocognition or social cognition this transfer can take place in different ways: 1) through homework tasks such as in *Recos* (*Remédiation Cognitive dans la Schizophrénie—Cognitive Remediation for Schizophrenia*) (25, 26) or *CRT* (*Cognitive Remediation Therapy*) programs (27, 28), social cognition programs such as *SCIT* (*Social Cognition Interaction Training*) (29) or *TomRemed* (*Remédiation en Théorie de l’Esprit—Theory of Mind Remediation*) (30) programs. 2) transfer to everyday life can also be facilitated through group sessions oriented toward full explanations of the cognitive domains and consequences in daily life, and explicit work on transfer of benefits such as in the *NEAR* (*Neuropsychological Educational Approach to Remediation*) program (31) or as we develop it in the Virtual Reality serious game program “*Jeu Mathurin*”, which trains planning abilities and prospective memory in a virtual town (17). Finally, participants in their rehabilitation trajectory could also experience other psychosocial programs: psychoeducation (32–34), management and support for their caregivers (such as the Canadian program *Profamille—Profamilly*) (32), Cognitive Behavioral Therapy (CBT) (35) or physical adapted activity program (36). All the CR programs are delivered in a standardized protocol: 1) multidisciplinary evaluation (medical, neuropsychological and functional), 2) feed-back to the user and eventually his family of his strengths and weaknesses, 3) psychoeducation session about neurocognitive or social cognition functions using a formalized handbook agreed by our national health agency (ETP 11106) 4) Questions about the handbook the users had to read carefully at home consecutively to the psychoeducation session. Then users and the C3RP team begin the CR sessions.

However, the crucial question is to see what the future is made of for participants enrolled in CR, several years after the end of the program. Therefore, a retrospective survey has been conducted to ask all of our participants about outcomes in terms of work, studies, but also clinical stability, functional environment, and the participant feelings about CR intervention. Our main assumption regarding our primary outcome was significant changes in terms of rate of employment or active student status between T1 (period of service delivery when the CR program took place) and since T2 (end of CR service delivery) at time of the follow-up survey. The survey was approved by the ethic committee (CPP Île-de-France VI, N° 2017-A00704-49).

## Materials and Methods

The survey included all the participants treated with CR since the creation of the unit from 2009 up to 2016. The survey was conducted in 2017–2018. Age of participants was ranged between 21 and 63. The DSM 5 (37) diagnosis of the users initially recruited in the C3RP was predominantly schizophrenia and schizoaffective disorder, with a scarce number of bipolar disorders (the C3RP was initially focused on rehabilitation in schizophrenia). Progressively were also admitted persons with autism or presenting complex neurodevelopmental disorder. The users were contacted by phone by the C3RP team and informed that they will receive by post or mail a survey including 12 main domains, which could include one to five sub domains, and questions relevant to some determinants of their actual professional and functional outcome, housing, relatedness, familiar relationships, daily activities. There were 19 questions and 15 sub-questions related to questions if there was a “yes” answer were asked (see **Table 1**). A consent form was sent, after explaining the study orally by the clinician and the patients had to sign it. Lastly, the Narrative Evaluation of Intervention Interview (NEII) (38, 39) was also sent to express feelings about CR. The questionnaire encompassed 15 total questions, with an equal number of questions related to the process and to the results of the intervention. Participants had to give their fully written consent to participate to this survey. As many patients did not return this survey, we proposed them to come to visit us and fulfill the documents in our unit.

**Table 1:** The survey questionnaire.

Survey Questions
Socio-demographic information
Years of CR
Before and after CR: job or no job employment, sheltered or not sheltered employment, studies, graduation.
Questions about the type of regular clinical follow-up they actually have (private or public visits to the psychiatrist)
Treatment users actually have (the treatment they had when they participated to the CR program was reported in the CR file)
If users regularly visit different type of French mental health units: day-care, therapeutic activities, day-life assistance. Also, users were asked about their putative registration in a club-house or a mutual help users associative group.
Leisure practice: sport, reading books, regular visits in libraries or others (verbatim).
Inner feeling of clinical stability, relapses, number of relapses, hospitalizations and if yes, the duration of these hospitalizations.
Questions about their private condition of living: independent housing or housing in the family, single or living as a couple, having children
Questions about participation to other psychosocial therapy programs: psychoeducation, programs for care-givers, cognitive behaviour therapy, physical adapted activity.

### Type of CR Programs Delivered

From 2009 to 2012 only two individual neurocognitive programs were available in the C3RP: *CRT* (27), and *RECOS* (25). In mid 2013, we introduced a French program focused on Theory of mind difficulties—*TOM-REMED* (30) -, and in 2014 the *Mathurin Serious Game* (17). Finally, since 2015 we delivered *NEAR* (31) and the *SCIT* programs (29). Hence, from 2014 the diversity of neurocognitive and social cognition methods provided a fully enriched panel of therapies with personalized rehabilitation course adapted to the cognitive profile of users.

### Statistical Analysis

Statistical analyses were performed using Jamovi (40) for quantitative data and Iramuteq (41) for qualitative data. First descriptive statistics were produced. Numerical variables were summarized as mean and standard deviation (SD), whereas counts and frequencies were used for categorical variables. In order to investigate the difference on patient characteristics (sociodemographic, diagnostic, medication), delivered services (type of CR programs and psychosocial therapies) and outcomes (professional activity, studies, housing, leisure, physical activity and relapse) between before service entry and in 2017–2018 since the end of care, McNemar Chi²-Test for categorical variables and paired sample t-test for numeric variables were performed. For comparison between the different time period of service delivery (2009–2013 and 2014–2016) Pearson Chi²-Test for categorical variables and independent sample t-test for numeric variables were performed. Lastly, we use a multivariate logistic regression model to identify potential predictors of primary outcome (employment or active student status since the end of T2 in 2017–2018). Concerning textual data with the NEII questionnaires, a lexicometric analysis using the Reinert method (42) was performed in order to identify different cluster of patient subjective evaluation of CR effects.

## Results

The survey included 131 P-RC, but only 66 completed the survey and signed their consent, and 52 returned the NEII. Three eligible persons for CR died by suicide, unrelated to the rehabilitation course, with two of them who experienced a CR program but died several years after. Initially, our purpose was to compare users eligible for CR who achieved the program (n = 92), to users who dropped-out after the initial evaluation (n = 40), to obtain two comparable groups in terms of indication for CR. Unfortunately, among the drop-out users, only one questionnaire was returned. Hence, we analyzed only the data of participants achieving the CR (P-CR) (see **Table 2**).

**Table 2:** Number of participants invited to fulfill the survey in reference to the starting year of cognitive remediation, completion and non-completion rates.

Cognitive Remediation	Survey Invitations 2017–2018	Drop-out Cognitive Remediation	Completed Cognitive Remediation	Survey Reponses*
Starting year	N	n (%)	n (%)	n (%)
**2009**	16	5 (31.3)	11 (68.7)	6 (37.5)
**2010**	16	2 (12.5)	14 (87.5)	10 (62.5)
**2011**	8	4 (50.0)	4 (50.0)	2 (25.0)
**2012**	14	4 (28.6)	10 (71.4)	8 (57.1)
**2013**	21	8 (38.1)	13 (61.9)	11 (52.4)
**2014**	22	3 (13.6)	19 (86.4)	12 (54.5)
**2015**	32	14 (43.8)	18 (56.2)	14 (66.7)
**2016**	3	0 (0.0)	3 (100.0)	3 (100.0)
**Total**	**131**	**40 (30.5)**	**92 (70.2)**	**66 (50.4)**

*All respondents completed their cognitive remediation program.

Socio-demographic characteristics of the P-CR, as well as diagnoses and T1-Since T2 pharmacological categories of treatments they received are listed in **Table 3A**. Distribution concerning the type of programs participants achieved, number of P-CR experiencing single or combination of CR methods as well as other psychosocial therapies are presented in **Table 3B**. When users were invited to different CR programs, this was done consecutively, with neurocognitive program first, followed by a social cognition program if necessary. Patient could have done previously or enter any psychosocial therapy program after having done a CR program in the C3RP unit. For the whole P-CR group the mean number of years with non-activity before CR was 4.2 with a standard deviation of 6.1.

**Table 3A:** Socio-demographic, diagnoses and T1-Since T2 treatments of the participants.

Cognitive Remediation	At T1 (Before CR)	T1-Since T2 (After RC)	p value*
	n or *mean* (% or *SD*)	n or *mean* (% or *SD*)	
Socio-Demographics			
Male Sex	41 (62.1)		
Age	*38,7 (93.9)*		
*Years of study*	*13,4 (2.9)*		
Diagnostics			
Schizophrenia	53 (80.3)		
Bipolar	3 (4.5)		
Neurodevelopmental Disorder	10 (15.2)		
Any Treatment			
Antipsychotics	62 (93.9)	63 (95.5)	0.317
Clozapin	23 (34.8)	25 (37.8)	0.564
Depot antipsychotics	8 (12.1)	9 (13.6)	0.564
Depot 1 injection/15 days	8 (12.1)	0 (0.0)	<0.001
*Equivalent chlorpromazine*	*210,2 (184.8)*	*226,5 (197.4)*	*0.421*
Antidepressants	30 (45.5)	26 (39.4)	0.317
Mood stabilizers	13 (19.7)	13 (19.7)	1.00
Benzodiazepins	7 (10.6)	10 (15.2)	0.366
Anxiolytics or hypnotics	12 (18.2)	3 (4.5)	0.020
Methylphenidate	2 (3.0)	3 (4.5)	0.317

*McNemar Chi-square Test or paired sample t test.

**Table 3B:** Panel of programs delivered in the CR center, number of patients who participated to these programs in single or combined course, and who experienced other psychosocial therapies.

Cognitive Remediation Programs	Reference	Principal method	n (%)
Remédiation Cognitive dans la schizophrénie (RECOS)	(25)	Neurocognition (Individual computer/paper/pencil	31 (47.0)
Cognitive Remediation Therapy (CRT)	(27)	Neurocognition (Individual paper/pencil)	28 (42.4)
Neuropsychological Educational Approach for Cognitive Remediation (NEAR)	(31)	Neurocognition (Group computer/bridging group)	8 (12.1)
Social Cognition Interaction Training (SCIT)	(29)	Social Cognition (Group)	5 (7.6)
Jeu Mathurin (JM)	(17)	Neurocognition (Group Virtual Reality)	3 (4,6)
Remédiation of Theory Of Mind (TOM-REMED)	(30)	Social Cognition (Group)	1 (1.5)
***Group format***			***13 (19.7)***
***Social cognition target***			***6 (9.1)***
**Single & combination**			
***Single***		***NC***	***58 (87.9)***
RECOS	(25)	NC	27 (40.9)
CRT	(27)	NC	25 (37.9)
NEAR	(31)	NC	6 (9.1)
JM	(17)	NC	1 (1.5)
***Combination***			***7 (10.6)***
CRT + RECOS	(25, 27)	NC + NC	1 (1.5)
RECOS + SCIT	(25, 29)	NC + SC	1 (1.5)
CRT + TOM-REMED	(27, 30)	NC + SC	1 (1,5)
NEAR + SCIT	(29, 31)	NC + SC	2 (3.0)
JM + RECOS + SCIT	(17, 25, 29)	NC + NC + SC	2 (3.0)
**Psychosocial therapies**			**25 (37.9)**
CBT	(35)		16 (24.2)
Patient Psycho-Education	(33, 34)		11 (16.7)
Family Psycho-Education	(32)		8 (12.1)
Adapted Physical Training	(36)		7 (10.6)

### Work or Study Outcome

Type of outcome results are presented in **Table 4**. A significant difference was found for job employment with more than half of P-CR being employed at since T2 (p > 0.001). Within job employment, competitive job (not specifically dedicated for persons with disabilities) also improved significantly with 36% of P-CR being with a competitive job since T2 (P = 0.007). Studying status also improved significantly between T1 and since T2 with 30.3% being with a student status since T2 (p = 0.033). Among this group, the proportion of subjects who enrolled in an open study curriculum (without adaptation or dedication to persons with disabilities) significantly improved with 25.8% P-CR at since T2 (0.013).

**Table 4:** Type of outcome (work, studies, housing, leisure and physical activity) listed at T1 and T2.

Type of outcomes	At T1 n (%)	Since T2 in 2017–2018 n (%)	p value*
**Professional activity (all types of jobs)**	19 (27.3)	39 (57.6)	<0.001
Open jobs	13 (19.7)	24 (36.4)	0.007
**Users performing studies (all type of studies)**	12 (18.2)	20 (30.3)	0.033
Users performing open studies	8 (12.1)	17 (25.8)	0.013
Living situation (Independent Housing)	21 (31.8)	41 (62.1)	0.066
Leisure (Reading)	9 (13.6)	28 (42.4)	0.002
Physical activity	12 (18.2)	34 (51.5)	0.033

*McNemar Chi-square test.

### Housing, Leisure and Physical Activity

When questioned about their leisure activities, a very significant difference was found between T1 and since T2 for P-CR who regularly red books or magazines, and a significant difference T1-Since T2 also existed for physical activity. At since T2, 62.1% of the group lived in an independent house, while the proportion was only 31.8% at T1.

### Predictors of Employment or Active Student

A logistic regression model, tested the influence of potential predictors such as age, sex, years of study, existence of relapses, treatment dosage (chlorpromazine equivalent), diagnostic, CR in group or participation in other psychosocial therapies, on employment or active student status since the end of T2 in 2017–2018. Only quantitative variable age and CPZ were significantly associated with a positive outcome (respectively 0.018 and 0.014). However, caution is advised in the interpretation since an increase of 1 unit in the respective quantitative variables represent a decrease of respectively 10% and less than 1% in the odds being employed or having an active student status (data not shown). In other words, the only predictors of a positive outcome were being younger and having a lower treatment dosage (43).

One important point was to know if the functional status was related to the time when users participated to the CR program. Therefore, we split the whole number of participants in two subgroups: subgroup 1: 2009–2013 (n = 37) and subgroup 2: 2014–2016 (n = 37). Characteristics of the two subgroups of P-CR are mentioned in **Table 5**. The split-year of the whole sample was 2014, because that year represented the initiation of an enriched panel of CR programs, with more group methods in neurocognition or social cognition proposed in the unit.

**Table 5:** Characteristics of P-CR who participated to CR programs between 2009 and 2013 (Subgroup1) and between 2014 and 2016 (Subgroup2).

Cognitive Remediation	2009–2013 (n = 37)	2014–2016 (n = 29)	p value*
	n or *mean* (% or *SD*)	n or *mean* (% or *SD*)	
**Socio-Demographics**			
Male Sex	20 (54.1)	21 (72.4)	0.048
*Age*	*41,3 (11.5)*	*34,0 (10.7)*	*0.041*
*Years of study*	*13,2 (2.9)*	*13,7 (2.7)*	*0.539*
**Diagnostics**			
Schizophrenia	34 (91.9)	19 (65.5)	
Bipolar	1 (2.7)	2 (6.9)	
Neurodevelopmental Disorder	2 (5.4)	8 (27.6)	0.026
**Programs**			
CR Combination	1 (2.7)	6 (20.7)	0.019
CR in Group	1 (2.7)	12 (41.4)	<0.001
CR Social cognition	1 (2.7)	5 (17.2)	0.041
Psychosocial Therapies	11 (29.7)	14 (48.3)	0.123
**Any Treatment at T2**	36 (97.3)	27 (93.1)	0.417
Antipsychotics	36 (97.3)	27 (93.1)	0.417
Clozapin	18 (48.7)	7 (24.2)	0.042
Depot antipsychotics	3 (8.1)	6 (20.7)	0.139
Depot 1 injection/15 days	0 (0.0)	0 (0.0)	0.325
*Equivalent chlorpromazine*	*259,4 (199.3)*	*184,5 (190.1)*	*0.127*
Antidepressants	17 (45.9)	9 (31.0)	0.219
Mood stabilizers	7 (18.9)	6 (20.7)	0.858
Benzodiazepins	5 (13.5)	5 (17.2)	0.675
Anxiolytics or hypnotics	2 (5.4)	1 (3.4)	0.705
Methylphenidate	2 (5.4)	1 (3.4)	0.705
**Type of outcomes since T2 (in 2017–2018)**			
Professional activity	26 (70.3)	13 (44.8)	0.037
Open jobs	18 (48.7)	6 (20.7)	0.019
Users performing studies	7 (18.9)	13 (44.8)	0.023
Users performing open studies	6 (16.2)	11 (39.3)	0.036
Independent Housing	23 (62.1)	18 (62.1)	0.994
Leisure (Reading)	16 (43.2)	12 (41.4)	0.879
Physical activity	21 (56.8)	13 (44.8)	0.336
No relapse	21 (56.8)	23 (79.1)	0.054

*Pearson Chi-square Test or independent sample t test.

When we examine socio demographical as well as clinical difference, subgroup 2 was younger than subgroup 1, with a higher proportion of males. Also, there were more persons with autism or complex neurodevelopment disorders. Considering the CR programs achieved, there was in subgroup 2 a higher proportion of combination of programs, of programs delivered in groups and of social cognition programs. The two subgroups did not differ for the number of other psychosocial therapies.

The type of outcome was also showing noticeable difference: there were significantly less P-CR in subgroup 2 having a professional activity and within them obtaining open jobs, but more users performing studies and among them open studies. Lastly, the number of no relapse was not significantly different in subgroup 1 and subgroup 2.

### Narrative Evaluation of CR Intervention Effects

For the whole sample a global evaluation of the narrative feelings using the NEII questionnaire (38, 39) is presented **Table 6**:

– Class 1 (41%) was referring to the incidence of CR on the functioning of thought: P-CR were describing more “clarity of thought”, more control of thought disorders, and better abilities to be attentive and to listen to others.
– Class 2 (30%) was around the effect of CR on concentration and memory associated to self-confidence. P-CR was describing better concentration, memory abilities, easiness to speak. They linked these improvement with gain in self-confidence.
– Class 3 (29%) was referring to work and studies. P-CR mentioned that CR helped for work and studies, and even recommended this therapy for persons “having health problems” or “problems with the treatment”.

**Table 6:** Global evaluation and lexicometric analysis of the narrative feeling written by the participants (n = 52).

Class	Main Topic	Most Prominent words	Most illustrative Verbatim
Class 1 (41%)	Incidence on the functioning of thought	thought; follow; feel; daily; test; organize; help	*Cognitive Remediation (CR) is a good help mainly to drive thought disorders* *This allows the development of attentive listening, of an active listening, an acute sense of observation, to establish a real dialog, to listen to the speaker and to the therapists; Also, it requires a constant effort to express correctly and clearly your thought.* *Acts on automatic thoughts, jumping to conclusions and recognize emotions of others.* *Clarity of thought as well as acuity for the selection of the terms and a refined spirit*.
Class 2 (30%)	Incidence on concentration and memory with an impact on self confidence	cognitive; task; concentration; enable; method; remediation; improvement	*CR was a real benefit, it allowed not only to work on my memory and concentration in a targeted way but also it gave me hope and confidence in abilities I thought were lost.* *The impact of CR for me are: rapidity to retrieve information, increase of concentration abilities, better memory, more fluent to speak, and more self- confidence* *CR brought many positive elements, it allowed to think to other things than making effort to be concentrated, less violent impulse, more self-confidence and a better quality in relationships.*
Class 3 (29%)	Incidence on studies and work	return; therapist; world; a follow-up;	*[recommend] to those who lost confidence in their abilities, or have difficulties in their studies consecutively to health problems or treatments effects* *I successfully returned studying and entered the professional world, and I think I cannot do better.* *I have been able to return to studies in a library and* *I am able to read books, because it’s stimulating and it’s a booster to make intellectual progress.*

## Discussion

This survey clearly shows that in a sample of participants who experienced personalized CR programs, a significant proportion of users obtain a job, with a high number of persons who are employed in open works, doing studies, reading or practicing physical activity regularly, when referring to their status or leisure before CR. When we examine the interval of 8 to 4 years on one hand, and 3 to 1 year on the other hand, from the survey-period, there was a significant number of persons who got a job in the former group, and a significant number of persons to come or return to studies in the latter group, with similar determinants for outcome and a high number of no relapse for these two subgroups.

Concerning employment, in the literature, only 11.5% of persons with a psychic handicap exert an open job while this proportion turns around 62.2% in the general population, despite the fact that 55 to 70% of users with psychic handicap claim they would like to work (44). In an European cohort of persons with schizophrenia enrolled in a naturalistic study with a 2-year follow-up the overall employment rate of participants was 21.5%, but varied between countries and sites, with rates of 12.9% in the UK, and 11.5% in France. During the same period the general population employment rate in France was 62.2% (45). However, in certain conditions such as in rural china compared to urban environment, high rates of employment can be seen for persons having a severe mental illness. In our study, when open and sheltered jobs are considered, the rate of employment we find is 57.6% with a significant proportion of persons who exerts a job after CR, in comparison to the proportion before CR. This rate is nearly the same as the range of rates for employment after Computer assisted CR done by outpatients with schizophrenia or schizoaffective disorders listed in the meta-analysis of Chan et al. (8) (54 to 69% depending on the different studies). This rate was significantly reduced in the subgroups of participants who received CR during 2014–2016 compared to the group treated during 2009–2013. For the former period the rate is 70.3%, with 48.7% doing ordinary jobs. As a matter of example, the Individual Placement and Support (IPS) method’s [Boardman and Rinaldi (1) listed in Pachoud and Corbière (46)], known as a particularly efficient method for supported employment, provides rates of open employment around 60%, compared with rates of around 25% obtained with the usual mental health services. In France, since 2016 an adaptation of the IPS method has been currently implemented all over the land. Our group of participants did not benefit from the pilot IPS experience beginning in 2016. Hence, we make the assumption that our CR personalized models for care added to the French IPS program should certainly reinforce the good outcome results for the users. In our group the percentage of users working in sheltered employment was 12%, while in the European cohort of Marwaha et al. (45), the same percentage in the French group was 30%. However, this cohort was collected before 2007 and psychosocial therapies were very scarce in France before 2009.

The overall number of users performing studies was also significantly different at T1 (18%) compared to T2 (30%), with also a higher number of users performing ordinary studies (36.4%). The noticeable point is that this number is significantly higher between 2014 and 2016, compared to the group doing CR in 2009–2013. However, the more recent group is younger. That could be part of the explanation of the higher proportion of users who obtained jobs in the former group, and the higher proportion of users performing studies after CR in the latter one. In a large group of persons with psychosis living in an Australian urban city, Jablensky et al. (47) found in a cohort of 980 individuals with schizophrenia 11.6% of persons achieving a tertiary education diploma degree, with 58.1% who had left school at age 16 years or earlier and 47.8% who completed secondary schooling. Moreover, plausibly our recruitment changed from 2009 to 2013 compared to 2014–2016 with younger participants to who psychosocial therapies have been proposed. Lastly, what is noticeable is that even before the enrichment of CR methods in 2014–2015, persons with neurocognitive deficits treated with tailored program of rehabilitation could find jobs and could maintain it after many years. During this earlier period persons were mainly treated with CRT and Recos, two neurocognitive methods. However, even if these two methods are focused on neurocognition, using CRT, Wykes et al. (48) found that for aged persons with schizophrenia, when they experienced a memory benefit, there was in parallel an improvement in social behavior. Finally using Recos, Vianin (49) insisted on the mixed paper/pencil and computer program which brings positive effects on metacognition with more transfer in everyday life.

At the moment of the survey, 92.4% of the P-RC were feeling as clinically stable. The number of untreated patients was quite the same between T1 and since T2, and the CPZ equivalent were also very similar in the two subgroups. This survey sample was mainly composed of participants with schizophrenia. In the literature, the rate of relapses in schizophrenia is variable. As a matter of example, a systematic review (50) found in multiple episodes of schizophrenia remission rates from 16 to 62%. Moreover, these rates depend from several factors. Comparing the different periods, 2009–2013 and 2014–2016, there was quite the same proportion of subjects who experienced no relapse after CR. This result is in favor of a continuing benefit of psychosocial therapy even after several years. Mueller et al. (51) recently found convergent results showing that the INT-CR-Program prevent relapses in a one year follow up study in schizophrenia. When we look to some determinants of global functioning, at T1, 18% of users were regularly practicing physical activity (including frequent walk), and after CR this proportion was significantly higher, with nearly half of the P-CR. Also, a higher proportion of users were reading regularly. Moreover, since T2, 62.1% had independent housing, while there were less users in the same environment at T1.

Generally, to scrutiny analyze all these results, a control group might be necessary and is lacking in our study. It has to be done in the future. However, when we reconcile these results with the high percentage of employment and studies in this sample, we could state that our users show in their outcomes several determinants that have been mentioned in studies about recovery (52). However, without specific recovery or symptom questionnaire or scales we cannot go forward in this hypothesis. Morin and Franck (53) states that clinical remission and overall functioning are two main factors for recovery. We did not find any impact of confounding factors on our findings except for age and treatment dosage that could be obviously understood as the lower the age and the level of symptomatology that needs to be treated with antipsychotics, the higher the chance is for a positive outcome, especially concerning employment or studies. However, Erim et al. (43) found in a group of persons with schizophrenia that a low dosage of antipsychotics was correlated with young age, shorter disease duration, symptomatic remission and higher rate of employment. Nevertheless, the small group of participants could raise insufficient power issues on analysis. Another point is the low rate of responses to the survey by the participants. This low rate has many explanations: 1) probably a low motivation of these drop-out subjects, who for some of them did not achieve the program or even did not begin the program after the baseline evaluation. 2) The long delay after the years of CR treatment could for other participants be also a reason of non-response. 3) The fact that persons come in our rehabilitation center from many parts of the very large Ile de France region. These persons could have changed their address, or even live in another region.

When looking carefully to the lexicometric analyses of the intervention evaluation extracted from the Narrative interview of the P-CR, three classes emerged, with three main topics (**Table 5**): one was concerning the thought functioning, with the positive impact on CR on clarity of thought and on benefits in driving thought disorders; the second topic establishes a link between the benefit of CR on cognitive functions and the association with self-confidence; and the third topic concerned the positive incidence of CR on work and studies. These topics are nodal objective of CR and the participants who responded fully perceive these effects. Confirmation of these benefits also come from literature: Farreny et al. (54) showed an association between improvement in executive functioning after CR and reduction of thought disorders. Seccomandi et al. (23) recently pointed out that the improvement in self-esteem might be a moderator of the response of CR with a link between higher self-esteem at baseline and better competitive employment as well as lower unemployment (55). However, in another study self-esteem had no influence on cognitive gains (16). Lastly, Bell et al. (56) demonstrated benefits of CR on supported employment for schizophrenia. Finally, participant subjective evaluation of CR effects converge with what experts of rehabilitation teams are expecting from this therapy. Interestingly, it was noticeable that “participants recommend this treatment for difficulties to study because of mental health problems.” Nowadays in France, CR for mental health difficulties is extensively growing all over the national territory. In each region rehabilitation centers have already been developed or are in an ongoing process of development. Every year, therapists are formally trained when they want to deliver CR programs. Our consortium for CR (Association Francophone de Remédiation Cognitive—AFRC- and therapy CR network) exists since 2009, and formal university graduations for CR and psychosocial rehabilitation exist in Lyon and Paris, while formal training concerning individual CR programs are delivered in a context of professional continuous training to nurses, psychologists, psychiatrists and other clinician stake holders. In the overall mental health policy in France, psychosocial therapies and mainly CR have been designed as national priorities for mental health (Instruction DGOS/R4/2019/10).

Lastly, more forms exist in the responses given by subgroup 2 than by subgroup 1. Obviously one can more easily retrieve precise and rich details about a therapy when memories are more recent.

### Limitations

This manuscript is a very preliminary study concerning long term outcome of a small number of persons. The main limitation of this study concern power issues and the absence of a control group which deeply limits the possibility to refer to a population of persons recruited during the same period in the same environment. Furthermore, outcomes might be measured on a different time scale for different subject since they’re not assessed at the end of CR but in 2017–2018 since the end of service delivery (from 2009 to 2016). Also, are lacking formal clinical evaluation, as well as questionnaires exploring satisfaction, recovery and self-report memories of the participants themselves concerning the feeling of recovery. Our sample is probably biased; one of the indirect probe for this bias is the number of patients eligible for CR who dropped out (30.5%) or did not answer to our survey (response rate of 50.4%). The selection bias was reinforced by our model for rehabilitation care: to enter in a CR program users have to be motivated, and must have an idea of the concrete project of insertion they want to concretize. However, in a context of our French free medical health insurance, one has to keep in mind the cost of psychosocial therapies in general; Hence, we must obtain a minimum of guarantee that programs could be followed until the end to prove that these psychosocial therapies must continue and need an extension in France.

## Conclusion

The main findings of this study highlight the plausible efficacy of personalized cognitive remediation in naturalistic conditions to promote overall functioning. Strikingly, these results are found even several years after the intervention and regardless of the time when it was applied, with a high percentage of participants who works after cognitive remediation in open jobs, who studies or who acquires training and graduation. Also, some determinants of overall functioning which are frequently expressed in recovery have also been pointed out. After cognitive remediation, inner feeling of increase of self-confidence, better clarity of thought, and feeling that cognitive remediation has been a real help for work, studies or mental health problems are directly expressed by the participants. Few relapses can be showed and these effects are maintained, even many years after the program. All these factors exist in a tailored care delivery for cognitive remediation and psychosocial therapies, in a precise timed course adjusted to the rehabilitation project, with huge efforts to transfer benefits of remediation in daily living, coordinated to the clinical follow-up of the sector team which continues to help the user in his rehabilitation project. However, to be confirmed undoubtedly these findings have to be done in reference to a control group. Also, a follow-up prospective study has to be carried on. Cognitive remediation and psychosocial rehabilitation seem to provide actually modest but robust improvement. Comparative studies reporting long term effect of this psychosocial therapy are warranted to confirm these preliminary findings.

## Data Availability Statement

The datasets generated for this study are available on request to the corresponding author.

## Ethics Statement

The studies involving human participants were reviewed and approved by CPP île de France VI. The patients/participants provided their written informed consent to participate in this study.

## Author Contributions

IA, MM, and YM wrote the manuscript. All the other authors contributed to collect the data for this survey, to correct the manuscript, and to supervise the study. YM and FP performed the statistical analyses. LK completed some statistical analyses and supervised the collection of data concerning psychosocial therapies.

## Funding

The study was promoted by the GHU Paris, Psychiatrie et Neurosciences. The C3RP receives a financial help from Pierre Deniker foundation.

## Conflict of Interest

The authors declare that the research was conducted in the absence of any commercial or financial relationships that could be construed as a potential conflict of interest.
